# Aortic morphological variability in patients with bicuspid aortic valve and aortic coarctation

**DOI:** 10.1093/ejcts/ezy339

**Published:** 2018-10-30

**Authors:** Froso Sophocleous, Benedetta Biffi, Elena Giulia Milano, Jan Bruse, Massimo Caputo, Cha Rajakaruna, Silvia Schievano, Costanza Emanueli, Chiara Bucciarelli-Ducci, Giovanni Biglino

**Affiliations:** 1 Bristol Medical School, University of Bristol, Bristol, UK; 2 Institute of Cardiovascular Science, University College London, London, UK; 3 Bristol Heart Institute, University Hospitals Bristol, NHS Foundation Trust, Bristol, UK; 4 Division of Cardiology, Department of Medicine, University of Verona, Verona, Italy; 5 Vicomtech-IK4, Data Intelligence for Energy and Industrial Processes, Donostia/San Sebastián, Spain; 6 Cardiorespiratory Division, Great Ormond Street Hospital for Children, NHS Foundation Trust, London, UK; 7 National Heart and Lung Institute, Imperial College London, London, UK

**Keywords:** Congenital heart disease, Bicuspid aortic valve, Aortic coarctation, Aorta, Magnetic resonance imaging, Computational modelling

## Abstract

**OBJECTIVES:**

This study aimed to explore aortic morphology and the associations between morphological features and cardiovascular function in a population of patients with bicuspid aortic valve, while further assessing differences between patients with repaired coarctation, patients with unrepaired coarctation and patients without coarctation.

**METHODS:**

This is a single-centre retrospective study that included patients with available cardiovascular magnetic resonance imaging data and native bicuspid aortic valve diagnosis (*n* = 525). A statistical shape analysis was performed on patients with a 3-dimensional magnetic imaging resonance (MRI) dataset (*n* = 108), deriving 3-dimensional aortic reconstructions and computing a mean aortic shape (template) for the whole population as well as for the 3 subgroups of interest (no coarctation, repaired coarctation and unrepaired coarctation). Shape deformations (modes) were computed and correlated with demographic variables, 2-dimensional MRI measurements and volumetric and functional data.

**RESULTS:**

Overall, the results showed that patients with coarctation tended towards a more Gothic arch architecture, with decreased ascending and increased descending aorta diameters, with the unrepaired-aortic coarctation subgroup exhibiting more ascending aorta dilation. Careful assessment of patients with repaired coarctation only revealed that a more Gothic arch, increased descending aorta dimensions and ascending aorta dilation were associated with reduced ejection fraction (*P* ≤ 0.04), increased end-diastolic volume (*P* ≤ 0.04) and increased ventricular mass (*P* ≤ 0.02), with arch morphology distinguishing patients with and without recoarctation (*P* = 0.05).

**CONCLUSIONS:**

A statistical shape modelling framework was applied to a bicuspid aortic valve population revealing nuanced differences in arch morphology and demonstrating that morphological features, not immediately described by conventional measurements, can indicate those shape phenotypes associated with compromised function and thus possibly warranting closer follow-up.

**Figure:**
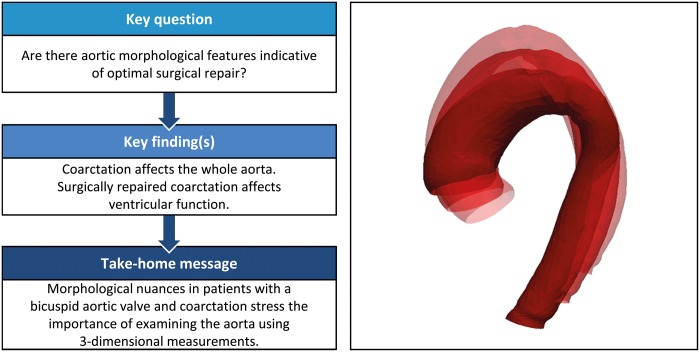

## INTRODUCTION

Bicuspid aortic valve (BAV) is the most common congenital heart abnormality, with 1–2% prevalence, and 3:1 predominance in males [1] and is often observed in association with other congenital defects such as aortic coarctation (CoA) or interrupted aortic arch. It is a clinically heterogeneous disorder, involving the aortic valve and 1 or more segments of the proximal ascending aorta, leading to valve stenosis/regurgitation and progressive aortic dilation [2]. Current knowledge of BAV disease is incomplete, especially with regard to the impact on segmental aortic morphology [3].

The clinical assessment of the aorta mostly relies on echocardiography or other cross-sectional imaging techniques, monitoring aortic diameters but failing to capture the full 3-dimensional (3D) variability in aortic shape, angles and size from patient to patient. The presence of dilation and/or CoA, the latter likely repaired with different strategies, can lead to further morphological variability, suggesting the need for 3D measurements to go beyond the simple dimensional assessment [4, 5]. The abundance of 3D information provided by medical imaging can be fully exploited using a novel statistical shape modelling (SSM) methodology to quantitatively evaluate the morphology of an entire vascular region of interest as a contiguous unit [4, 6, 7]. Refined understanding and better characterization of the anatomy as a 3D domain can provide insight into flow dynamics, complications associated with vascular geometry and disease progression [8].

This study aimed to explore nuances in 3D aortic morphology in a BAV population. We hypothesized that BAV patients with and without CoA exhibit overall different arch architecture. The study also aimed to explore the morphological features of repaired CoA in relation to shape and function, revealing possible functional differences underlying different approaches to CoA repair, considering that the clinical significance of CoA in BAV aortopathy is not fully understood [9]. We hypothesized that BAV patients with CoA present a more Gothic arch architecture and worse function.

## MATERIALS AND METHODS

### Patient population

**Figure 1:**
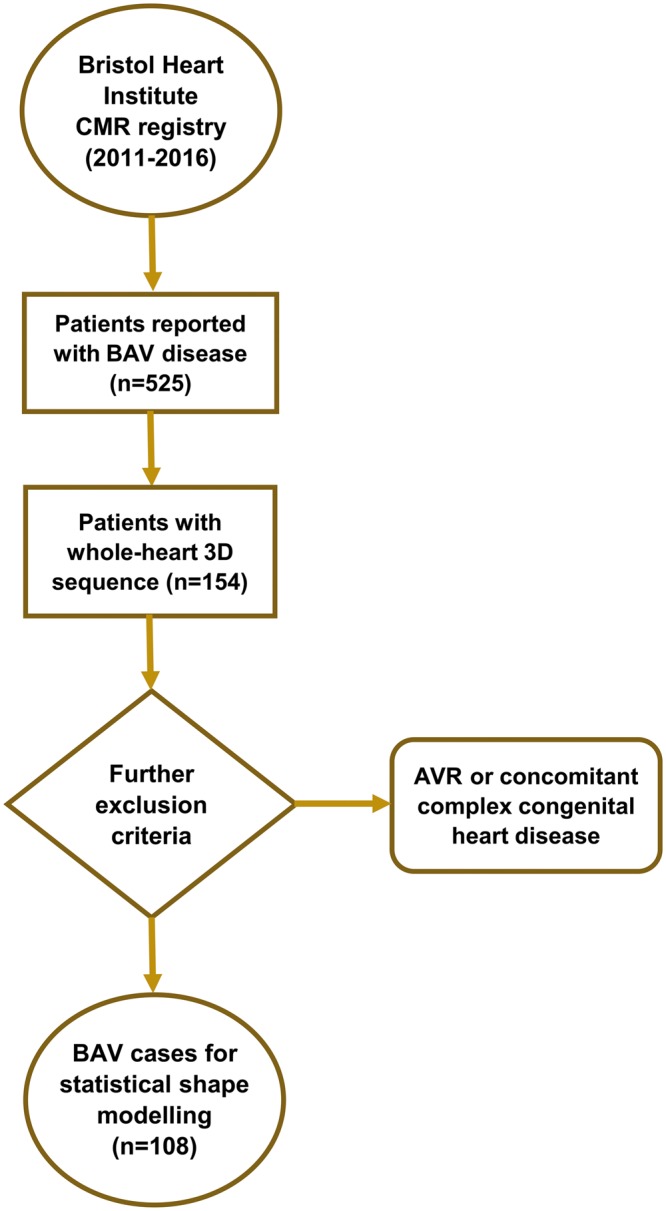
Patient selection. Inclusion/exclusion criteria: presence of 3D sequence (*n* = 154), valve morphology (*n* = 17), complex congenital heart diseases (*n* = 2), aortic valve replacement (*n* = 7), ascending aortic replacement (*n* = 4), Ross procedure (*n* = 9) and arch reconstruction (*n* = 2). 3D: 3-dimensional; AVR: aortic valve replacement; BAV: bicuspid aortic valve; CMR: cardiovascular magnetic resonance.

This was a retrospective single-centre study. Consecutive BAV patients (*n* = 525) were identified in the cardiovascular magnetic resonance (CMR) imaging database between 2011 and 2016; 154 patients had CMR data suitable for 3D reconstructions, i.e. MR angiogram. Exclusion criteria included aortic valve replacement (AVR), aortic root reconstruction, unconfirmed bicuspid morphology and comorbities including complex congenital heart defects involving some form of aortic reconstruction (e.g. Norwood procedure). A final population (*n* = 108) was thus obtained (Fig. 1), including 5 patients with 2 scans. CMR data were acquired at 1.5 T (Avanto, Siemens Healthineers, Erlangen, Germany). Demographic variables were collected from clinical patient records. Anatomical and functional variables were collected from CMR reports. These include left ventricular (LV) volumes and ejection fraction, aortic valve morphology classified according to the coronary fusion pattern, presence of aortic valve dysfunction, including aortic regurgitation and aortic stenosis, and presence or absence of CoA and reCoA. Aortic regurgitation was classified according to the regurgitant fraction as mild (≤30%), moderate (31–49%) or severe (≥50%) [10]. Aortic stenosis was classified according to the valve area measured by planimetry as mild (>1.5 cm^2^), moderate (1.0–1.5 cm^2^) and severe (<1.0 cm^2^) [10]. In the absence of stenosis and regurgitation, valves were classified as normofunctioning. Aortic coarctation was diagnosed in the presence of anatomical significant narrowing of the descending aorta in the region of the isthmus. In patients with known history of CoA repair, reCoA was defined as a recurrence of narrowing in the coarctation site. The severity of anatomical narrowing was classified according to the ratio of the CoA diameter to the descending thoracic aorta and defined as severe if ≤0.5 and absent if ≥0.85 [11]. Ultimately, the population was divided into 3 subgroups: patients with isolated BAV and no coarctation (*n* = 37), patients with BAV and repaired aortic coarctation (*n* = 58) and patients with BAV and primary unrepaired coarctation (*n* = 13).

All datasets were anonymized and, in view of the retrospective study design, formal ethical approval was waived by the local Institutional Research and Innovation Department.

### Creation of 3-dimensional models and aortic measurements

**Figure 2:**
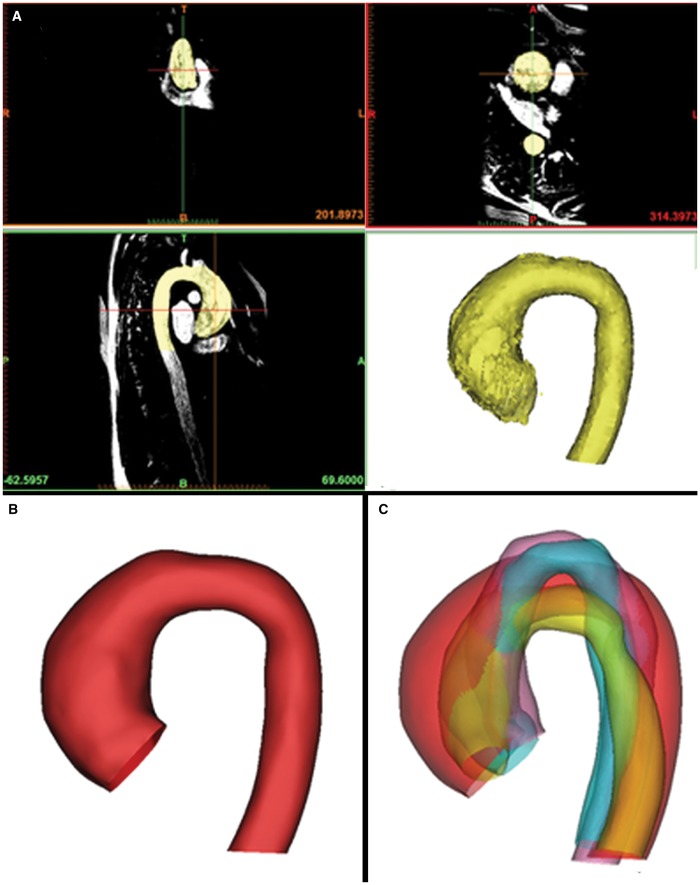
An overview of the preprocessing steps prior to a shape analysis. The ascending aorta, the transverse aortic arch and the descending aorta cut at the subannular level and at the level of the diaphragm were manually segmented from 3-dimensional imaging data (**A**). The segmented models were cut, meshed and smoothed (**B**). All the shape models were rigidly registered (**C**).

**Figure 3:**
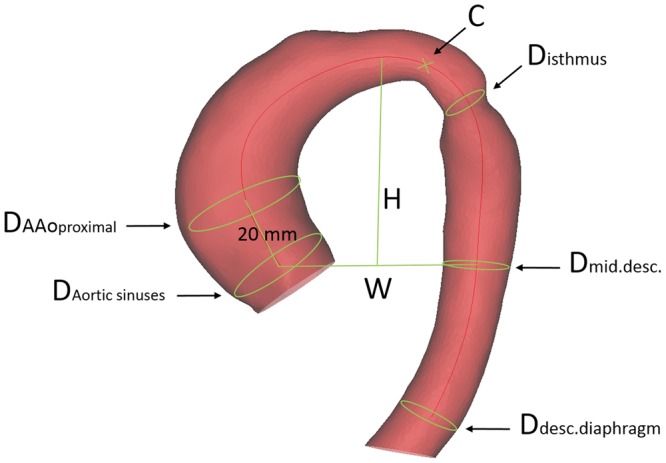
Measurements were taken manually on each 3-dimensional shape model, including the centreline curvature, the aortic arch width and height, the diameter of the aorta at the sinuses, 2 cm above the sinotubular junction, at the isthmus, the mid-descending aorta and the diaphragm. The curvature of the centreline was measured, indexing over the patient’s height [8]. The aortic arch width was measured as the perpendicular distance from the centreline at the sinotubular junction level to the centreline at the mid-descending aorta. The height of the arch was measured as the maximum vertical distance from the width measurement to the highest point of the centreline in the arch, calculating an *H*/*W* ratio as a surrogate for Gothic arch architecture. AAo: ascending aorta; D: diameter; desc.: descending aorta; H/W: height/width.

CMR images provided the input data for the SSM, generating 3D aortic volumes for all 108 cases (Fig. 2A) using commercial software (Mimics Research v.19.0, Materialise NV, Leuven, Belgium). The 3D aortic models were consistently cut perpendicularly at the subannular level and at the level of diaphragm (Vascular Modelling Toolkit, Orobix, Bergamo, Italy) (Fig. 2B). The brachiocephalic, left common carotid, left subclavian and coronary arteries were excluded because the study focused on examining the aorta alone. An aortic centreline was calculated (resolving resolution and distance between control points set at 3.125 mm, smooth factor 0.5). Aortic diameters around the centreline were measured at the aortic sinuses, the ascending aorta, the isthmus and the descending aorta (Fig. 3). In addition to aortic diameters, other anatomical measurements performed on the 3D aortic arch models included aortic curvature [8, 12–14], aortic arch width (*W*) and height (*H*) and arch tortuosity. The latter was calculated as [1 − (*W*/*l*)] where *l* is the incremental length of the centreline between the points defining aortic width [4, 13].

### Details of image analysis and statistical shape modelling

LV and aortic valve anatomical, functional and volumetric assessments, as well as thoracic aortic measurements were performed using an image post-processing package (Circle Cardiovascular Imaging, Calgary, Canada).

The mean aortic shape in the population and variations around this mean were computed using SSM [4]. The models were uniformly remeshed and smoothed (3matic Research v.11.0, Materialise), exported as computational surface meshes and automatically aligned (i.e. rigidly registered) to reduce possible bias due to differences in translation and rotation during SSM (Fig. 2C). The aligned 3D arch surface models represent the SSM input. An SSM framework was used to process and visualize the 3D shape information, avoiding the need for landmarking or point-to-point correspondence [4, 15]. Deformetrica software (www.deformetrica.org) was used to compute the average aortic shape, or ‘template’, from all the registered 3D aortic shape models of each individual patient. The same software was used to compute the deformation vectors from the template towards each patient’s specific shape, quantifying the variation of each patient from the mean aortic shape of the population [16]. The combined set of patient-specific deformation vectors describes all 3D shape variability in the population. Principal component analysis was applied to the computed deformation vectors, reducing the complex 3D shape variation to few components or ‘shape modes’. Shape modes represent specific aspects of the anatomical variation of the aorta and help to understand the morphological characteristics that cannot be described by aortic diameters alone. Video 1 shows an example of a shape mode from our population that describes aortic arch angulation. The contribution of each mode is visualized deforming the template from low [−2.7 standard deviation (SD)] to high (+2.7 SD) values of each mode’s deformation vector (Video 1). Shape modes can not only be visually displayed but also numerically quantified by shape vectors that numerically represent the contribution that each shape mode has on each subject and were used for statistical analyses [5] thus supporting the identification of specific shape features (e.g. aortic dilation, Gothic arch architecture and increased tortuosity).

### Statistical analysis

Statistical analysis (R, Vienna, Austria) included demographic factors, CMR-derived volumetric parameters, traditional morphometric measurements and computed shape modes, across repaired, unrepaired and no-CoA subgroups. Depending on the distribution of the continuous variables, the *t*-test or the Mann–Whitney test was used. Differences across the 3 subgroups were tested with either one-way analysis of variance or Kruskal–Wallis tests. Differences between categorical variables were assessed using the *χ*^2^ test. We explored associations between shape modes and demographic, volumetric or traditional morphometric parameters using univariate and multivariate regression analysis. A *P*-value <0.05 indicated statistical significance. Analyses were repeated for repaired-CoA patients to identify changes within this subgroup of interest.

## RESULTS

Demographic, clinical, valve anatomical and functional data and thoracic aortic measurements are reported in Table 1, together with differences in aortic measurements. Valve function significantly differs among the 3 subgroups with 24% of normofunctioning valves in patients with no-CoA, 52% in the repaired-CoA subgroup and 23% for the unrepaired-CoA subgroup (*P* = 0.01). No significant difference was observed in LV volumes and left ventricular ejection fraction (LVEF) across the 3 subgroups, whilst LV mass was significantly increased in the unrepaired-CoA subgroup. The repaired-CoA subgroup was heterogeneous and previous repair included end-to-end anastomosis (*n* = 31), subclavian flap (*n* = 11), stenting (*n* = 4), patch (*n* = 3) and graft (*n* = 4), and a proportion of patients (*n* = 11) also received ballooning of the CoA. Age of CoA repair was 7 ± 8 years (range 0–33). A large proportion of patients with CoA (51/71) had known history of hypertension.

**Table 1:** Demographic, clinical and functional variables and traditional morphometric measurements

	No-CoA	Repaired-CoA	Unrepaired-CoA	*P*-value
Variables				
Patients (*n*)	37	58	13	
Gender (*n* males)	26	33	10	*P* = 0.2
Age (years)	47.2 ± 18.6	33.9 ± 12.2	30.1 ± 16.6	*P* < 0.001
BSA (m^2^)	1.85 ± 0.2	1.9 ± 0.2	1.8 ± 0.2	*P* = 0.05
LVEF (%)	64.6 ± 6.8	62.6 ± 7.4	66.9 ± 8.8	*P* = 0.4
LVEDV (ml)	87.6 ± 20.9	84.5 ± 18.5	93.6 ± 36.9	*P* = 0.9
LV mass (g)	76.9 ± 24.3	64.2 ± 15.6	78.9 ± 31.7	*P* = 0.04
Aortic stenosis (*n*, %)	14, 38	13, 22	3, 23	*P* = 0.2
Severity of aortic stenosis (*n*, %)				
Mild	6, 43	8, 62	1, 33	
Moderate	2, 14	2, 15	1, 33	
Severe	6, 43	3, 23	1, 33	
Aortic regurgitation (*n*, %)	23, 62	27, 47	9, 69	*P* = 0.2
Severity of aortic regurgitation (*n*, %)				
Mild	14, 61	23, 85	7, 78	
Moderate	4, 17	3, 11		
Severe	5, 22	1, 4	2, 22	
Normal AV function (*n*, %)	9, 24	30, 52	3, 23	*P* = 0.01
Fusion pattern (*n*, %)				*P* = 0.5
Right–left	29, 78	50, 86	10, 77	
Right/left-non-coronary	8, 22	8, 14	3, 23	
Measurements				
Aortic sinuses (mm)	39.6 ± 7.6	32.9 ± 6.4	33.9 ± 9.6	*P* = 0.9
AAoproximal (mm)	39.9 ± 9.1	31.6 ± 6.7	34.2 ± 11	*P* < 0.001
Desc. mid. (mm)	22.9 ± 0.2	22.4 ± 5.7	22.3 ± 9.3	*P* = 0.3
Desc. diaphragm (mm)	21.7 ± 4.5	20.5 ± 4.5	19.9 ± 7.9	*P* = 0.1
*H* (mm)	65.4 ± 19	61.8 ± 14.6	71.5 ± 23	*P* = 0.2
*W* (mm)	77.3 ± 16.2	66.8 ± 12	65.7 ± 18.4	*P* = 0.001
*H*/*W*	0.9 ± 0.2	0.9 ± 0.2	1.1 ± 0.2	*P* = 0.005
*C* (mm^−1^)	0.05 ± 0.01	0.07 ± 0.02	0.07 ± 0.02	*P* < 0.001
*C*/*H*_pt_ (mm/m^2^)	16.9 ± 6.5 × 10^−3^	22.7 ± 7.6 × 10^−3^	23.6 ± 7.4 × 10^−3^	*P* < 0.001
Isthmus (mm)		17.4 ± 3.9	13.8 ± 6	*P* = 0.06
CoA index		0.9 ± 0.2	0.7 ± 0.2	*P* = 0.05
*T* (mm)	0.6 ± 0.1	0.6 ± 0.08	0.7 ± 0.08	*P* = 0.008

Values are represented as mean ± standard deviation.

AAo: ascending aorta; AV: aortic valve; BSA: body surface area; *C*: curvature; CoA: coarctation of the aorta; Desc.: descending aorta; *H*/*W*: height-to-width ratio; *H*: aortic height; *H*_pt_: patient’s height; LV: left ventricular; LVEDV: left ventricular end-diastolic volume; LVEF: left ventricular ejection fraction; *T*: tortuosity; *W*: width.

**Figure 4:**
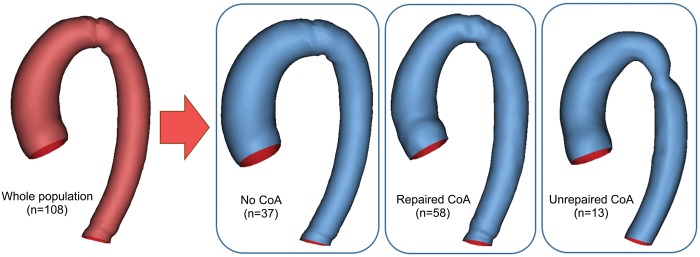
Aortic templates: whole population, no-CoA, repaired-CoA and unrepaired-CoA. CoA: coarctation of the aorta.

Aortic templates were calculated for the whole population and for the 3 subgroups (Fig. 4). The qualitative assessment of the templates revealed increased height and decreased width for both the repaired- and unrepaired-CoA groups compared to the no-CoA group. The unrepaired-CoA template had greater curvature, suggesting a prominent Gothic aortic arch (Fig. 4). Patients with isolated BAV and no coarctation had significantly larger ascending aortic dimensions compared to the other subgroups (39.9 ± 9.1 vs 31.5 ± 6.7 mm for the repaired-CoA group and 34.2 ± 10.9 mm for the unrepaired-CoA group), as well as significantly decreased curvature and increased width, suggesting a less Gothic aorta. Aortic tortuosity was associated with the presence of CoA (*P* = 0.02) and differed significantly between subgroups (*P* = 0.008). The repaired and unrepaired-CoA subgroups had higher tortuosity, unrepaired-CoA patients being even more tortuous.

**Figure 5:**
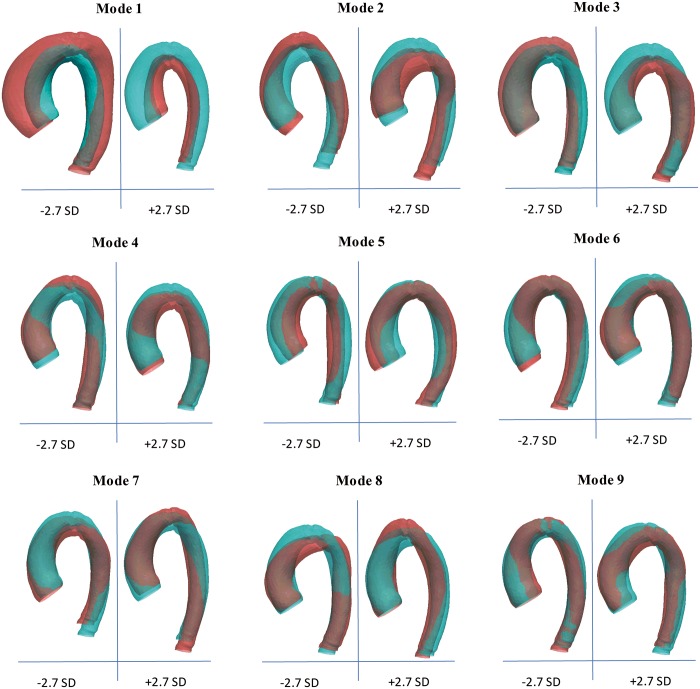
Three-dimensional shape features in the whole population, showing dominant shape modes as deformations of the computed template from low (−2.7 SD) to high (+2.7 SD) values. Blue shapes indicate template and red shapes indicate mode extremities. SD: standard deviation.

The first 9 principal component analysis shape modes (Fig. 5) recapitulated 72% of the overall shape variability in the population (Supplementary Material, Table SA), and hence the corresponding shape vectors were used for statistical analyses. Different modes captured different morphological features, after careful visual assessment and correlation with traditional morphometric measurements (Supplementary Material, Table SB). Dominant shape features of interest included overall aortic size (mode 1), ascending aorta dimensions (modes 2, 3, 5, 6, 7 and 9), descending aorta dimensions (modes 2, 3, 5, 6 and 7), angulation/Gothic arch (modes 2, 3, 4, 5, 6 and 8), coarctation (modes 3, 6 and 7) and tortuosity (modes 2, 3, 5, 6 and 8). Overall, significant differences were found for modes 1 (*P* = 0.007), 2 (*P* = 0.02), 3 (*P* < 0.001), 4 (*P* = 0.002), 6 (*P* = 0.03) and 8 (*P* = 0.02), suggesting gross as well as detailed changes across the 3 shape phenotypes of interest. Aortic size was overall significantly smaller in patients with CoA; older age (*P* < 0.001), higher body surface area (*P* < 0.001) and male gender (*P* < 0.001) were significantly associated with increased aortic size (mode 1) in univariate analysis, and all remained significant when tested in a multivariate model.

The no-CoA subgroup approached a rounder arch architecture (modes 2, 3, 4 and 8) with decreased height (modes 2, 4, 6 and 8) and increased width (modes 2, 6 and 8), decreased tortuosity (modes 2, 3, 6 and 8) and more substantial ascending aortic dilation (modes 2, 3 and 6). The aortic arch morphology in the repaired-CoA subgroup tended towards a more Gothic aortic arch architecture (modes 2, 4 and 8) with increased height (modes 2, 4, 6 and 8) and increased tortuosity (modes 2, 3, 6 and 8), less dilated ascending aorta (modes 2, 3 and 6) but increased diameters of the mid-descending aorta (modes 2, 3, 6 and 8) and at the level of the diaphragm (modes 3, 4 and 6). The unrepaired-CoA subgroup presented similar features to the repaired CoA subgroup, i.e. more Gothic aortic arch architecture (modes 2, 6 and 8) and increased tortuosity (modes 2 and 8), with slightly more dilated ascending aorta (modes 3 and 6).

**Figure 6:**
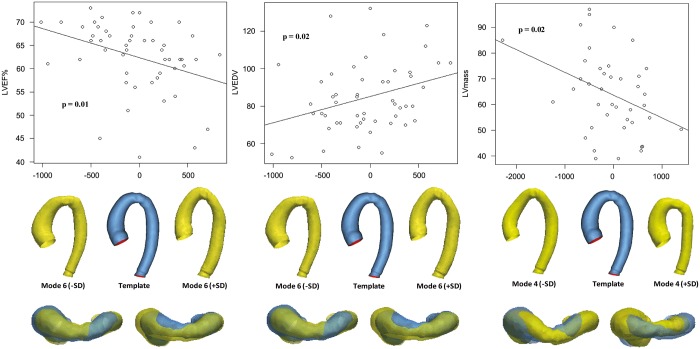
Correlations between mode 6 and LVEF (*left*), mode 6 and LVEDV (*centre*) and mode 4 and LV mass (*right*), showing front and top views of mode extremities. Patients with lower LVEF, higher LVEDV and higher LV mass tend towards mode extremities showing a more Gothic arch and increased tortuosity, decreased ascending and increased descending aortic diameter. LV: left ventricular; LVEDV: left ventricular end-diastolic volume; LVEF: left ventricular ejection fraction; SD: standard deviation.

Focusing on the repaired-CoA subgroup, 28 patients presented some degree of recoarctation (graded as mild in 16 and moderate in 12 patients). Shape modes and vectors specific for the repaired-CoA subgroup were also successfully computed. Dominant shape features in this subgroup describe aortic size (mode 1), ascending aorta dimensions (modes 5, 6, 7 and 9), descending aorta dimensions (mode 3–7), aortic architecture/Gothic arch (modes 2–9), coarctation (modes 5 and 6) and tortuosity (modes 2 and 5). Correlations between modes and anatomical, volumetric and functional data are reported in detail in Supplementary Material, Tables SB and SC. Associations between mode 4 and LV mass, modes 2 and 6 and LVEF, and modes 4 and 6 and left ventricular end-diastolic volume (LVEDV) suggested an ‘unfavourable aortic configuration’, whereby patients approaching a more Gothic and tortuous arch architecture with smaller ascending and larger descending aorta diameters tended to have lower LVEF (some <50%), higher LVEDV and higher LV mass (Fig. 6).

The presence of reCoA of any degree was associated with modes 6, 7 and 8, and it was also associated with a more Gothic arch architecture, but not with tortuosity or other morphometric/demographic variables. As expected, it correlated significantly with a smaller CoA index (*P* = 0.002).

## DISCUSSION

This study applied an SSM framework to elucidate 3D aortic morphology in the presence of BAV with and without aortic coarctation. The association between BAV and coarctation is well known, with BAV occurring in up to three-fourth of patients with coarctation; however, it is not clear how these disorders contribute to the morphology and pathology of the entire aorta [2, 9]. Therefore, a more detailed assessment of the aorta in these patients is needed to better capture the morphological features of interest [17] and potentially shed light on disease progression. SSM results showed that the presence of CoA affects the whole architecture of the aorta and that the CoA is not a localized disease of the proximal descending aorta but affects the aorta in its entirety.

A 3D SSM allows one to extract unique shape modes that visually and numerically represent complex shape features that are otherwise difficult to capture with traditional morphometric measurements. As the shape modes are deformations of the average shape in a population, it is reasonable that complex dominant shape features, such as aortic dilation (i.e. aortic root and proximal ascending aorta dilation) and arch architecture (i.e. Gothic versus round), are recapitulated by more than 1 mode. This reaffirms the need for a more detailed 3D analysis. Moreover, whilst the first modes (e.g. modes 1 and 2) represent overall changes in shape and recapitulate a bigger percentage of shape variability, other modes may contribute towards more local changes that should also be considered. This consideration guided the selection of the first 9 modes for analysis. Qualitative and quantitative findings suggested that patients with CoA tend to have Gothic architecture, which is verified by the increased height-to-width ratio and increased curvature, in agreement with the literature [4, 5, 9, 13]. Furthermore, arch tortuosity was higher in patients with repaired-CoA compared to patient without CoA, suggesting that arch morphology is different even in the presence of repair.

The presence of BAV in patients with coarctation increases the risk of aneurysm formation, with a tendency to develop aneurysm in 1:2 cases compared to 1:4 in coarctation patients without BAV [18]. According to a retrospective analysis [19] that analyses a cohort of repaired and unrepaired-CoA patients with (*n* = 142) and without (*n* = 74) BAV, ascending aortic dilation was mainly present in BAV patients and aortic valve dysfunction was sparse. In our study, we treated the repaired and unrepaired-CoA subgroups separately to identify possible changes in aortic morphology. Our repaired-CoA subgroup tended towards increased descending rather than ascending aorta diameters, confirming previous observations [4]. Additionally, both the unrepaired-CoA and no-CoA subgroups had higher LV mass and valve dysfunction compared to the repaired-CoA subgroup. An association (either compensatory or maladaptive) exists between high LV mass and aortic dysfunction [20, 21]. However, it is worth mentioning that an unfavourable functional implication of Gothic arch architecture has not always been consistently observed [22].

Mostly diagnosed and corrected during childhood, CoA is repaired but not necessarily cured [23]. Long-term complications after CoA repair include restenosis, pseudoaneurysm formation and descending aorta dilation and can lead to reintervention in up to 11% of cases [24, 25]. Prior CoA repair is likely to protect against ascending aortic dilation progression [26], but surveillance of the aorta in patients with BAV and CoA is warranted even in the presence of a good repair [18]. Within our population of patients with repaired coarctation, 45% presented any degree of reCoA at the time of the CMR scan. A closer look at the repaired CoA indeed showed stronger association with descending rather than ascending aorta dilation. In addition, our findings indicated that there is a specific aortic arch morphology in patients with repaired-CoA, a Gothic arch with increased descending aorta diameters that is associated with worse LV function, as also reported in the literature [5]. Furthermore, reCoA patients showed a tendency towards increased aortic dimensions.

Despite on-going efforts to assess the best type of surgical procedure regarding the formation of reCoA and late complications [27, 28], a link between aortic morphology and repair type has not been identified yet. In this population, increased tortuosity was associated with the presence and severity of CoA, and increased aortic tortuosity has been associated with aortic dilation, valve disease and unfavourable haemodynamics in the literature [29, 30]. So, in a theoretical search for optimal aortic configuration after CoA repair, it would appear that a less Gothic, less tortuous aorta with decreased descending aorta and/or ascending aorta diameters might be more favourable in terms of ventricular functional parameters.

**Figure 7:**
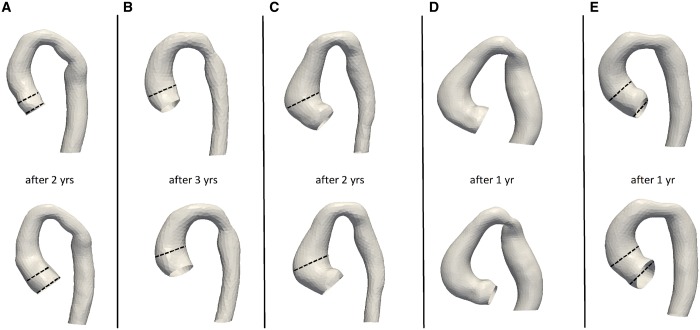
Aortic remodelling over time in patients with a follow-up whole-heart CMR scan over the 5-year study period. The dashed lines emphasize the increase in aortic diameter over time, in patients with CoA repair and/or reCoA (**A**, **B**, **C** and **E**), whereas in the patient with unrepaired CoA (**D**), this did not appear to increase.

A final observation was made on 5 patients who had 2 shapes reconstructed from CMR (i.e. 2 follow-up scans). Four of them (Fig. 7A, B, C and E) had undergone surgical CoA repair and showed dilation in the aortic root and/or ascending aorta, which was more pronounced in 3 cases with signs of reCoA (Fig. 7A, B and E). The patient without coarctation repair has unvaried ascending aortic dimensions as indicated both from qualitative assessment (Fig. 7D) and in the CMR reports. This preliminary observation on few isolated cases in our population can provide the basis for the application of this method to a growth/remodelling analysis, studying in detail how dimensions change over time, which will necessitate longitudinal data on a larger cohort. It should also be noted that patients with 2 scans were treated as 2 separate time points in the overall analysis, in consideration of possible changes in morphology (e.g. ascending aortic dilation), to be developed further in future studies using longitudinal data.

The study has the disadvantages of a retrospective design, with an uneven number of patients in the subgroups. A 3D sequence was a necessary requirement for the analysis but is not part of the routine imaging protocol for BAV at our centre; this might partly explain the high proportion of patients with CoA. Blood pressure data (cuff pressure at the time of CMR) were not available, and observations on hypertension are lacking but should be included in future prospective analyses. Furthermore, the repaired-CoA subgroup was heterogeneous, and further subgroup analysis between repair types could not be performed considering the small sample size in some of the subgroups. In order not to inflate Type II error of not detecting actual effects, computed correlation significances were not adjusted for multiple comparisons and all results are considered as exploratory.

## CONCLUSION

In conclusion, this study highlights nuances in aortic architecture between patients with/without coarctation, stressing the importance of considering the whole aorta in its 3-dimensionality. Aortic coarctation is likely not a localized disease but rather a disease of the whole aorta, which results in an overall change in the aortic arch morphology, and operated coarctation continues to be a disease of the aorta with repercussions on ventricular function. Whilst arch configuration after CoA repair is not necessarily under the control of the surgical team, the idea of an ‘optimal surgical shape’ should be further assessed in future studies, exploring aortic remodelling over time in BAV patients, adding a longitudinal perspective to the current cross-sectional design to further elucidate the morphological determinants of functional outcomes and possibly better informing patient follow-up.

## Funding

The authors acknowledge the support of the British Heart Foundation. This work was supported, and Chiara Bucciarelli-Ducci is partly funded, by the National Institute for Health Research Biomedical Research Centre at University Hospitals Bristol NHS Foundation Trust and the University of Bristol. The views expressed in this publication are those of the author(s) and not necessarily those of the NHS, the National Institute for Health Research or the Department of Health and Social Care.

**Conflict of interest:** Dr Chiara Bucciarelli-Ducci is a consultant for Circle Cardiovascular Imaging (Calgary, Canada). No other conflict of interest to declare.

## Supplementary Material

Supplementary Tables

Supplementary Video

## References

[1] Mordi I, Tzemos N. Bicuspid aortic valve disease: a comprehensive review. Cardiol Res Pract 2012;196037. 22685681 10.1155/2012/196037 PMC3368178

[2] Losenno KL, Goodman RL, Chu MW. Bicuspid aortic valve disease and ascending aortic aneurysms: gaps in knowledge. Cardiol Res Pract 2012;2012:145202. 23198270 10.1155/2012/145202 PMC3503270

[3] Siu SC, Silversides CK. Bicuspid aortic valve disease. J Am Coll Cardiol 2010;55:2789–800. 20579534 10.1016/j.jacc.2009.12.068

[4] Bruse JL, McLeod K, Biglino G, Ntsinjana HN, Capelli C, Hsia TY; Modeling of Congenital Hearts Alliance Collaborative Group. A statistical shape modelling framework to extract 3D shape biomarkers from medical imaging data: assessing arch morphology of repaired coarctation of the aorta. BMC Med Imaging 2016;16:40. 27245048 10.1186/s12880-016-0142-z PMC4894556

[5] Bruse JL, Khushnood A, McLeod K, Biglino G, Sermesant M, Pennec X et al Modeling of Congenital Hearts Alliance Collaborative Group. How successful is successful? Aortic arch shape after successful aortic coarctation repair correlates with left ventricular function. J Thorac Cardiovasc Surg 2017;153:418–27. 27776913 10.1016/j.jtcvs.2016.09.018

[6] Bruse JL, Cervi E, McLeod K, Biglino G, Sermesant M, Pennec X et al Modeling of Congenital Hearts Alliance Collaborative Group. Looks do matter! Aortic arch shape after hypoplastic left heart syndrome palliation correlates with cavopulmonary outcomes. Ann Thorac Surg 2017;103:645–54. 27592606 10.1016/j.athoracsur.2016.06.041

[7] Mansi T, Voigt I, Leonardi B, Pennec X, Durrleman S, Sermesant M et al A statistical model for quantification and prediction of cardiac remodelling: application to tetralogy of Fallot. IEEE Trans Med Imaging 2011;30:1605–16. 21880565 10.1109/TMI.2011.2135375

[8] Ntsinjana HN, Capelli C, Biglino G, Cook AC, Tann O, Derrick G et al 3D morphometric analysis of the arterial switch operation using *in vivo* MRI data. Clin Anat 2014;27:1212–22. 25156444 10.1002/ca.22458

[9] Roos-Hesselink JW, Scholzel BE, Heijdra RJ, Spitaels SE, Meijboom FJ, Boersma E et al Aortic valve and aortic arch pathology after coarctation repair. Heart 2003;89:1074–7. 12923033 10.1136/heart.89.9.1074 PMC1767804

[10] Lancellotti P, Tribouilloy C, Hagendorff A, Moura L, Popescu BA, Agricola E et al European Association of Echocardiography recommendations for the assessment of valvular regurgitation. Part 1: aortic and pulmonary regurgitation (native valve disease). Eur J Echocardiogr 2010;11:223–44. 20375260 10.1093/ejechocard/jeq030

[11] Lim DS, Ralston MA. Echocardiographic indices of Doppler flow patterns compared with MRI or angiographic measurements to detect significant coarctation of the aorta. Echocardiography 2002;19:55–60. 11884255 10.1046/j.1540-8175.2002.00055.x

[12] Ou P, Bonnet D, Auriacombe L, Pedroni E, Balleux F, Sidi D et al Late systemic hypertension and aortic arch geometry after successful repair of coarctation of the aorta. Eur Heart J 2004;25:1853–9. 15474701 10.1016/j.ehj.2004.07.021

[13] Ou P, Mousseaux E, Celermajer DS, Pedroni E, Vouhe P, Sidi D et al Aortic arch shape deformation after coarctation surgery: effect on blood pressure response. J Thorac Cardiovasc Surg 2006;132:1105–11. 17059930 10.1016/j.jtcvs.2006.05.061

[14] Ferreira Martins JD, Thomas B, Jalles Tavares N, Pinto FF. Aortic arch geometry after aortic coarctation repair: systematic magnetic resonance study in a consecutive series of patients. Rev Port Cardiol 2012;31:403–4. 22494835 10.1016/j.repc.2011.10.012

[15] Durrleman S, Pennec X, Trouve A, Ayache N. Statistical models of sets of curves and surfaces based on currents. Med Image Anal 2009;13:793–808. 19679507 10.1016/j.media.2009.07.007

[16] Durrleman S, Prastawa M, Charon N, Korenberg JR, Joshi S, Gerig G et al Morphometry of anatomical shape complexes with dense deformations and sparse parameters. Neuroimage 2014;101:35–49. 24973601 10.1016/j.neuroimage.2014.06.043 PMC4871626

[17] Yetman AT, Graham T. The dilated aorta in patients with congenital cardiac defects. J Am Coll Cardiol 2009;53:461–7. 19195601 10.1016/j.jacc.2008.10.035

[18] Preventza O, Livesay JJ, Cooley DA, Krajcer Z, Cheong BY, Coselli JS. Coarctation-Associated aneurysms: a localized disease or diffuse aortopathy. Ann Thorac Surg 2013;95:1961–7. 23643549 10.1016/j.athoracsur.2013.03.062

[19] Clair M, Fernandes SM, Khairy P, Graham DA, Krieger EV, Opotowsky AR et al Aortic valve dysfunction and aortic dilation in adults with coarctation of the aorta. Congenit Heart Dis 2014;9:235–43. 23764014 10.1111/chd.12109

[20] Kupari M, Turto H, Lommi J. Left ventricular hypertrophy in aortic valve stenosis: preventive or promotive of systolic dysfunction and heart failure? Eur Heart J 2005;26:1790–6. 15860517 10.1093/eurheartj/ehi290

[21] Ou P, Celermajer DS, Jolivet O, Buyens F, Herment A, Sidi D et al Increased central aortic stiffness and left ventricular mass in normotensive young subjects after successful coarctation repair. Am Heart J 2008;155:187–93. 18082512 10.1016/j.ahj.2007.09.008

[22] De Caro E, Trocchio G, Smeraldi A, Calevo MG, Pongiglione G. Aortic arch geometry and exercise-induced hypertension in aortic coarctation. Am J Cardiol 2007;99:1284–7. 17478158 10.1016/j.amjcard.2006.12.049

[23] Maia MM, Aiello VD, Barbero-Marcial M, Ebaid M. Coarctation of the aorta corrected during childhood. Clinical aspects during follow-up. Arq Bras Cardiol 2000;74:167–80. 10904291 10.1590/s0066-782x2000000200008

[24] Burkhart HM, Connolly H, Brown M, Dearani JA, Cetta F, Li Z et al Coarctation of the aorta: life-long surveillance is mandatory following surgical repair. J Am Coll Cardiol 2009;53:A361. 10.1016/j.jacc.2013.06.016 23850909

[25] Brown ML, Burkhart HM, Connolly HM, Dearani JA, Hagler DJ, Schaff HV. Late outcomes of reintervention on the descending aorta after repair of aortic coarctation. Circulation 2010;122:S81–4. 20837930 10.1161/CIRCULATIONAHA.109.925172

[26] Verma S, Siu SC. Aortic dilatation in patients with bicuspid aortic valve. N Engl J Med 2014;370:1920–9. 24827036 10.1056/NEJMra1207059

[27] Celermajer DS, Greaves K. Survivors of coarctation repair: fixed but not cured. Heart 2002;88:113–14. 12117824 10.1136/heart.88.2.113 PMC1767208

[28] Omeje I, Poruban R, Sagat M, Nosal M, Hraska V. Surgical treatment of aortic coarctation. Images Paediatr Cardiol 2004;6:18–28. 22368639 PMC3232524

[29] Doyle BJ, Norman PE, Hoskins PR, Newby DE, Dweck MR. Wall stress and geometry of the thoracic aorta in patients with aortic valve disease. Ann Thorac Surg 2018;105:1077–85. 29288661 10.1016/j.athoracsur.2017.11.061

[30] Morris SA. Arterial tortuosity in genetic arteriopathies. Curr Opin Cardiol 2015;30:587–93. 26398550 10.1097/HCO.0000000000000218 PMC4624847

