# Supplementary material for: Aortic morphological variability in patients with bicuspid aortic valve and aortic coarctation
Source: Eur J Cardiothorac Surg. 2018 Oct 30;55(4):704–13. doi: 10.1093/ejcts/ezy339 (PMC6459283; doi:10.1093/ejcts/ezy339)
Supplement: Supplementary Tables [file ezy339_supplementary_tables.docx]

**Supplementary Tables**

**Table A:** Overall contribution of the first nine modes to shape variability as % inertia.

| Modes | Inertia [%] | Cumulated Inertia [%] |
| --- | --- | --- |
| 1 | 24 | 24 |
| 2 | 12 | 37 |
| 3 | 8 | 44 |
| 4 | 7 | 51 |
| 5 | 6 | 57 |
| 6 | 5 | 62 |
| 7 | 4 | 66 |
| 8 | 4 | 70 |
| 9 | 3 | 72 |

**Table B:** Correlations between shape modes and traditional morphometric measurements.

| Whole Population Modes | Sinuses size | Proximal AAo | Mid. Desc. | Desc. Diaphragm | H | W | H/W | C | CoA Index | T |
| --- | --- | --- | --- | --- | --- | --- | --- | --- | --- | --- |
| 1 | p<0.001*** | p=0.3 | p<0.001*** | p<0.001*** | p<0.001*** | p<0.001*** | p=0.8 | p<0.001*** | p=0.9 | p=0.8 |
| 2 | p=0.07 | p=0.3 | p=0.02* | p=0.2 | p<0.001*** | p=0.5 | p<0.001*** | p=0.1 | p=0.9 | p<0.001*** |
| 3 | p<0.001* | p=0.2 | p=0.003** | p=0.1 | p=0.02* | p=0.8 | p=0.01* | p=0.8 | p=0.005** | p=0.05* |
| 4 | p=0.2 | p=0.8 | p=0.4 | p=1 | p=0.6 | p=0.4 | p=0.9 | p=0.3 | p=0.3 | p=0.6 |
| 5 | p=0.03* | p=1 | p=0.06 | p=0.007** | p=0.05* | p<0.001*** | p=0.005** | p=0.3 | p=0.8 | p=0.004** |
| 6 | p=0.08 | p=0.7 | p=0.02* | p<0.001*** | p=0.04* | p=0.08 | p=0.003** | p=0.8 | p=0.03* | p<0.001*** |
| 7 | p=0.1 | p=0.8 | p=0.09 | p=0.06 | p=0.3 | p=0.7 | p=0.1 | p=0.09 | p=0.01* | p=0.3 |
| 8 | p=0.4 | p=0.6 | p=0.4 | p=0.3 | p=0.02* | p=0.7 | p<0.001*** | p<0.001*** | p=0.2 | p=0.03* |
| 9 | p=0.1 | p=0.9 | p=0.7 | p=0.5 | p=0.9 | p=0.7 | p=0.3 | p=0.2 | p=0.2 | p=0.8 |
| Repaired-CoA  Modes | **Sinuses size** | **Proximal AAo** | **Mid. Desc.** | **Desc. Diaphragm** | **H** | **W** | **H/W** | **C** | **CoA Index** | **T** |
| 1 | p<0.001*** | p<0.001*** | p<0.001*** | p<0.001*** | p<0.001*** | p<0.001*** | p=0.7 | p<0.001*** | p=0.6 | p=1 |
| 2 | p=0.2 | p=0.7 | p=0.1 | p=1 | p<0.001*** | p=0.7 | p<0.001*** | p=0.7 | p=0.2 | p<0.001*** |
| 3 | p=0.2 | p=0.08 | p=0.001** | p=0.01* | p=0.07 | p=0.5 | p=0.02* | p=0.6 | p=0.3 | p=0.08 |
| 4 | p=0.4 | p=0.4 | p=0.03* | p<0.001*** | p=0.005** | p=0.004** | p=0.4 | p=0.1 | p=0.9 | p=0.4 |
| 5 | p=0.2 | p=0.1 | p=0.8 | p=0.8 | p=0.9 | p<0.001*** | p=0.003** | p=0.2 | p=0.003** | p<0.001*** |
| 6 | p=0.03* | p=0.1 | p=0.04* | p=0.001** | p=0.08 | p=0.8 | p=0.5 | p=0.1 | p<0.001*** | p=0.7 |
| 7 | p=0.05* | p=0.2 | p=0.009** | p=0.01* | p=0.5 | p=0.3 | p=0.3 | p=0.2 | p=0.8 | p=0.3 |
| 8 | p=0.3 | p=0.2 | p=0.1 | p=0.7 | p=0.02* | p=0.8 | p=0.004** | p=0.04* | p=0.9 | p=0.9 |
| 9 | p=0.02* | p=0.3 | p=0.4 | p=0.8 | p=0.3 | p=0.01* | p=0.2 | p=0.2 | p=0.5 | p=0.6 |

AAo=ascending aorta, Desc.=descending aorta, H= aortic height, W=width, H/W=height-to-width ratio, C=curvature, T=tortuosity. ***<0.001, **=0.001-0.009, *=0.01-0.05

**Table C:** Correlations between shape modes and demographic, clinical and functional variables.

| Whole Population  Modes | LVEF | LVEDV | LV mass | Aortic stenosis | Aortic regurgitation | Fusion pattern | CoA |
| --- | --- | --- | --- | --- | --- | --- | --- |
| 1 | p=0.3 | p=0.2 | p<0.001*** | p=0.02* | p=0.005** | p=0.7 | p=0.002** |
| 2 | p=0.03* | p=0.06 | p=0.04* | p=0.04* | p=0.9 | p=0.01* | p=0.006** |
| 3 | p=0.2 | p=0.2 | p=0.7 | p=0.1 | p=0.5 | p=0.7 | p<0.001*** |
| 4 | p=0.002** | p=0.1 | p=0.4 | p=0.2 | p=0.5 | p=0.04* | p<0.001*** |
| 5 | p=0.003** | p=0.2 | p=0.4 | p=0.8 | p=0.3 | p=0.5 | p=0.03* |
| 6 | p=0.7 | p=1 | p=0.07 | p=0.09 | p=0.8 | p=0.006** | p=0.02* |
| 7 | p=0.9 | p=0.3 | p=0.8 | p=0.9 | p=0.2 | p=0.1 | p=0.8 |
| 8 | p=0.6 | p=0.3 | p=0.2 | p=0.03* | p=0.06 | p=0.4 | p=0.02* |
| 9 | p=0.7 | p=0.03* | p=0.4 | p=0.05* | p=0.4 | p=0.9 | p=0.8 |
| Repaired-CoA  Modes | **LVEF** | **LVEDV** | **LV mass** | **Aortic stenosis** | **Aortic regurgitation** | **Fusion pattern** | **Re-CoA** |
| 1 | p=0.7 | p=0.06 | p=0.001** | p=0.05* | p=0.002** | p=0.2 | p=0.7 |
| 2 | p=0.04* | p=0.3 | p=0.06 | p=0.5 | p=0.6 | p=0.4 | p=0.7 |
| 3 | p=0.4 | p=1 | p=0.7 | p=0.5 | p=0.8 | p=0.2 | p=1 |
| 4 | p=0.1 | p=0.04* | p=0.02* | p=0.2 | p=0.1 | p=0.7 | p=0.8 |
| 5 | p=0.2 | p=0.2 | p=0.2 | p=0.8 | p=0.9 | p=0.3 | p=0.1 |
| 6 | p=0.01* | p=0.02* | p=0.8 | p=0.2 | p=0.7 | p=0.8 | p=0.05* |
| 7 | p=0.4 | p=0.8 | p=0.9 | p=0.3 | p=0.7 | p=0.8 | p=0.05* |
| 8 | p=0.5 | p=0.7 | p=1 | p=0.6 | p=0.4 | p=0.1 | p=0.05* |
| 9 | p=0.8 | p=0.8 | p=0.7 | p=0.6 | p=0.5 | p=0.02* | p=0.9 |

BSA=body surface area, LVEF=left ventricular ejection fraction, LVEDV=left ventricular end diastolic volume, CoA=presence of coarctation, Re-CoA=re-coarctation. ***<0.001, **=0.001-0.009, *=0.01-0.05
